# Supplementary material for: MyLynch: A Patient-Facing Clinical Decision Support Tool for Genetically-Guided Personalized Medicine in Lynch Syndrome
Source: Cancers (Basel). 2023 Jan 6;15(2):391. doi: 10.3390/cancers15020391 (PMC9856567; doi:10.3390/cancers15020391)
Supplement: Supplementary file 1 [file cancers-15-00391-s001.zip › MyLynch_supplemental_material.pdf]

Supplemental Material

December 23rd, 2022

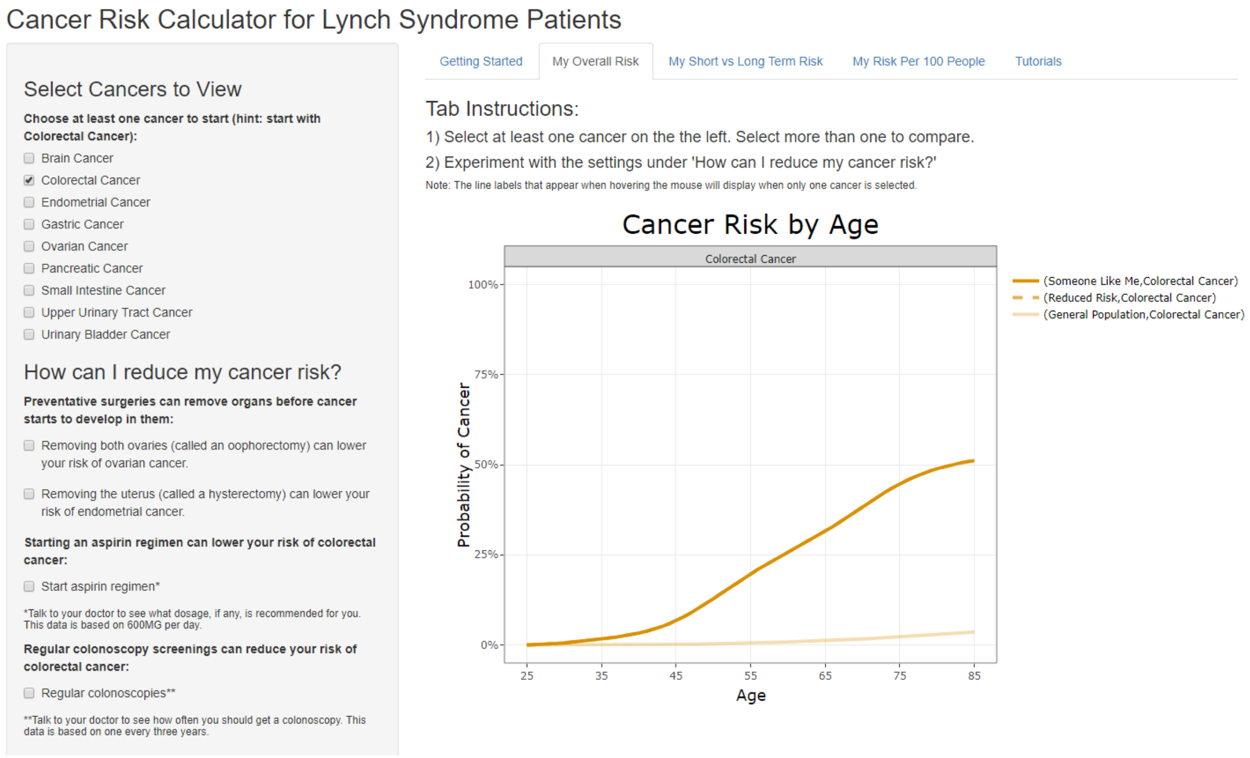

Figure S1: Screenshot from the minimum viable product produced as a result of the focus group feedback. The settings and risk information are for a hypothetical user.

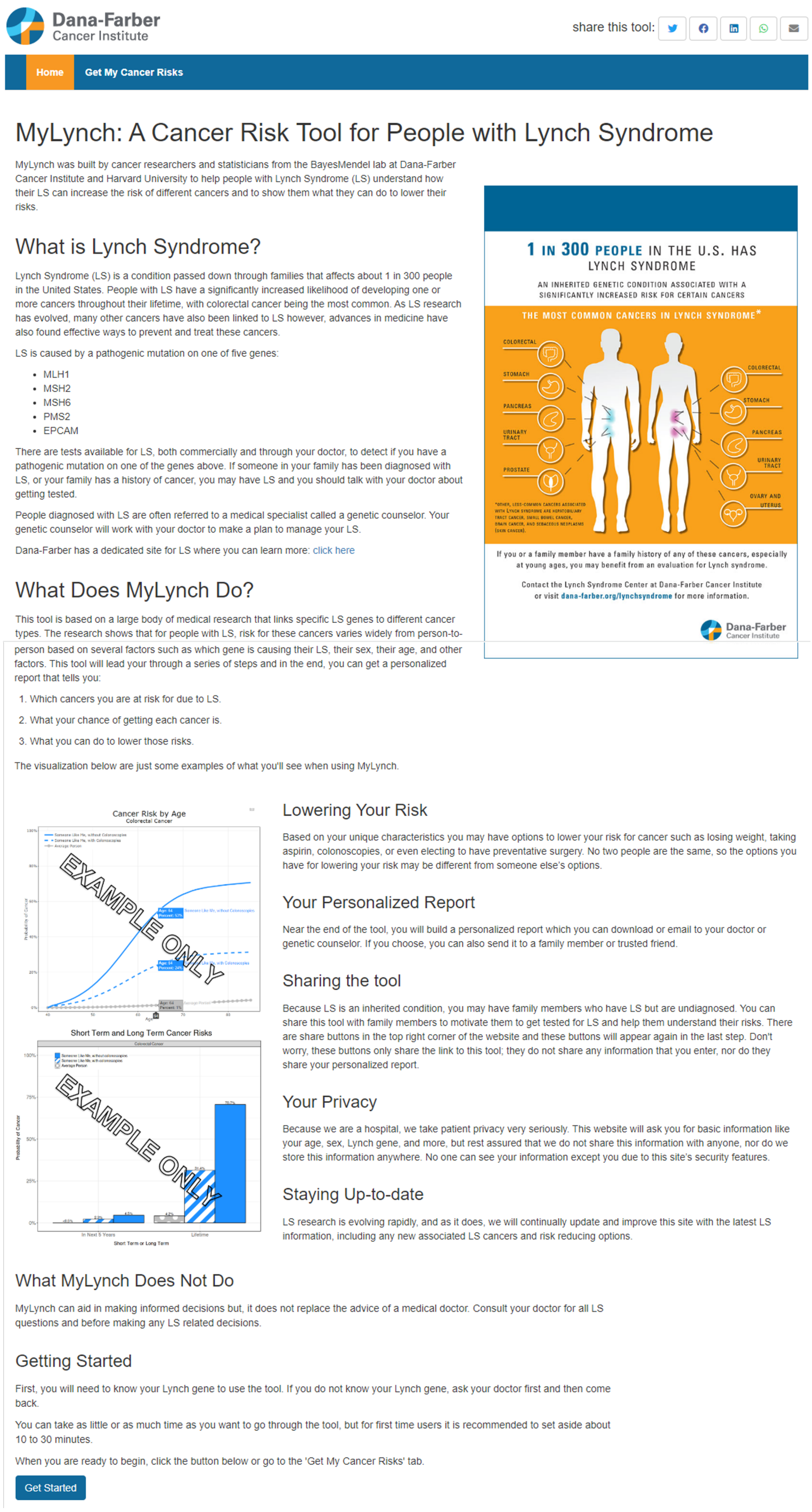

Dana-Farber Cancer Institute

share this tool:

Home | Get My Cancer Risks

MyLynch: A Cancer Risk Tool for People with Lynch Syndrome

MyLynch was built by cancer researchers and statisticians from the BayesMendel lab at Dana-Farber Cancer Institute and Harvard University to help people with Lynch Syndrome (LS) understand how their LS can increase the risk of different cancers and to show them what they can do to lower their risks.

What is Lynch Syndrome?

Lynch Syndrome (LS) is a condition passed down through families that affects about 1 in 300 people in the United States. People with LS have a significantly increased likelihood of developing one or more cancers throughout their lifetime, with colorectal cancer being the most common. As LS research has evolved, many other cancers have also been linked to LS however, advances in medicine have also found effective ways to prevent and treat these cancers.

LS is caused by a pathogenic mutation on one of five genes:

- MLH1
- MSH2
- MSH6
- PMS2
- EPCAM

There are tests available for LS, both commercially and through your doctor, to detect if you have a pathogenic mutation on one of the genes above. If someone in your family has been diagnosed with LS, or your family has a history of cancer, you may have LS and you should talk with your doctor about getting tested.

People diagnosed with LS are often referred to a medical specialist called a genetic counselor. Your genetic counselor will work with your doctor to make a plan to manage your LS.

Dana-Farber has a dedicated site for LS where you can learn more: click here

What Does MyLynch Do?

This tool is based on a large body of medical research that links specific LS genes to different cancer types. The research shows that for people with LS, risk for these cancers varies widely from person-to-person based on several factors such as which gene is causing their LS, their sex, their age, and other factors. This tool will lead your through a series of steps and in the end, you can get a personalized report that tells you:

1. Which cancers you are at risk for due to LS.
2. What your chance of getting each cancer is.
3. What you can do to lower those risks.

The visualization below are just some examples of what you'll see when using MyLynch.

Lowering Your Risk

Based on your unique characteristics you may have options to lower your risk for cancer such as losing weight, taking aspirin, colonoscopies, or even electing to have preventative surgery. No two people are the same, so the options you have for lowering your risk may be different from someone else's options.

Your Personalized Report

Near the end of the tool, you will build a personalized report which you can download or email to your doctor or genetic counselor. If you choose, you can also send it to a family member or trusted friend.

Sharing the tool

Because LS is an inherited condition, you may have family members who have LS but are undiagnosed. You can share this tool with family members to motivate them to get tested for LS and help them understand their risks. There are share buttons in the top right corner of the website and these buttons will appear again in the last step. Don't worry, these buttons only share the link to this tool; they do not share any information that you enter, nor do they share your personalized report.

Your Privacy

Because we are a hospital, we take patient privacy very seriously. This website will ask you for basic information like your age, sex, Lynch gene, and more, but rest assured that we do not share this information with anyone, nor do we store this information anywhere. No one can see your information except you due to this site's security features.

Staying Up-to-date

LS research is evolving rapidly, and as it does, we will continually update and improve this site with the latest LS information, including any new associated LS cancers and risk reducing options.

What MyLynch Does Not Do

MyLynch can aid in making informed decisions but, it does not replace the advice of a medical doctor. Consult your doctor for all LS questions and before making any LS related decisions.

Getting Started

First, you will need to know your Lynch gene to use the tool. If you do not know your Lynch gene, ask your doctor first and then come back.

You can take as little or as much time as you want to go through the tool, but for first time users it is recommended to set aside about 10 to 30 minutes.

When you are ready to begin, click the button below or go to the 'Get My Cancer Risks' tab.

Get Started

Figure S2: Screenshot from the homepage of MyLynch.

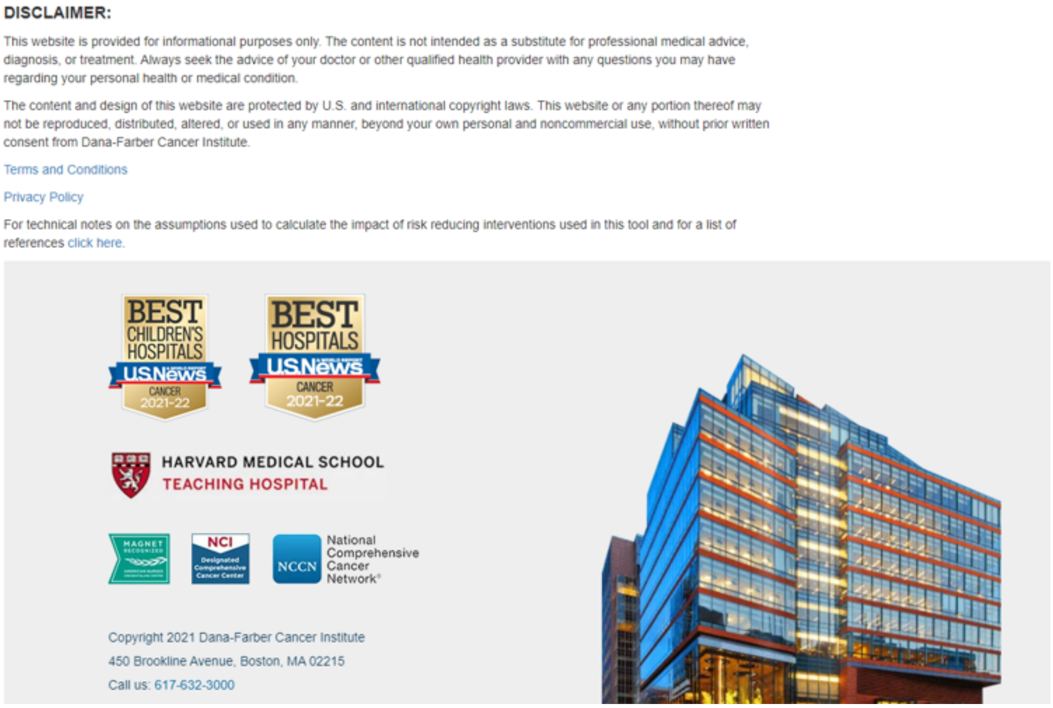

Figure S3: The footer displayed on every page of MyLynch which contains a disclaimer and links to the terms and conditions, privacy policy, and technical notes.

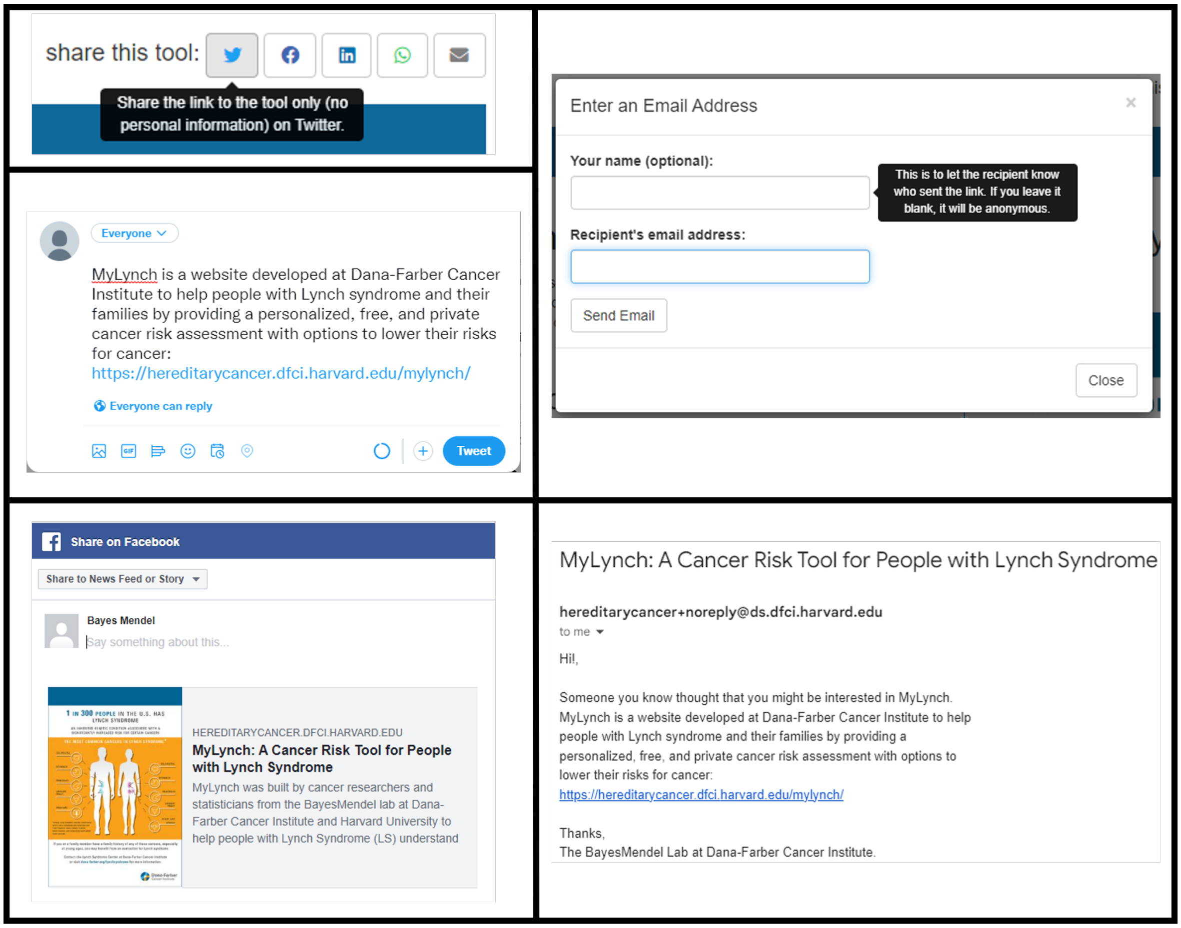

Figure S4: Several screenshots of sharing utility features for sending the link to MyLynch on various communication platforms. Top left: screenshot of then share buttons for Twitter, Facebook, LinkedIn, WhatsApp, and Email displayed at the top of every page; hover text that provides additional context is shown for the Twitter button; similar text is shown for all five buttons. Middle left: screenshot of the share message generated from the Twitter share button; the same text is used for WhatsApp. Bottom left: screenshot of the draft Facebook post/message generated by the Facebook share button; a similar draft post/message in generated in LinkedIn when that button is clicked; Facebook and LinkedIn do not allow text to be auto-populated in draft posts and messages, unlike Twitter and WhatsApp. Top right: screenshot of the pop-up window in MyLynch that appears when the email share button is clicked; using this window, the user can enter the desired recipient's email address and, optionally, the user can provide their name so that the recipient knows who sent the message, or they can send it anonymously. Bottom right: screenshot of the email generated by the email share button. Note: MyLynch does not track any information about the utilization of the share buttons, website redirects from embedded links, the user's name, nor the recipient's email address.

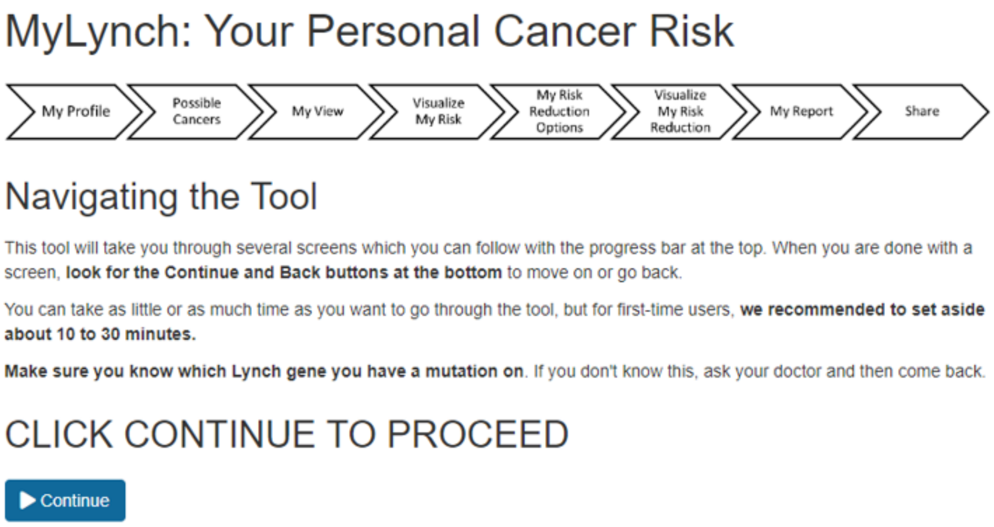

Figure S5: Screenshot of the instructions the user receives right after they view the homepage.

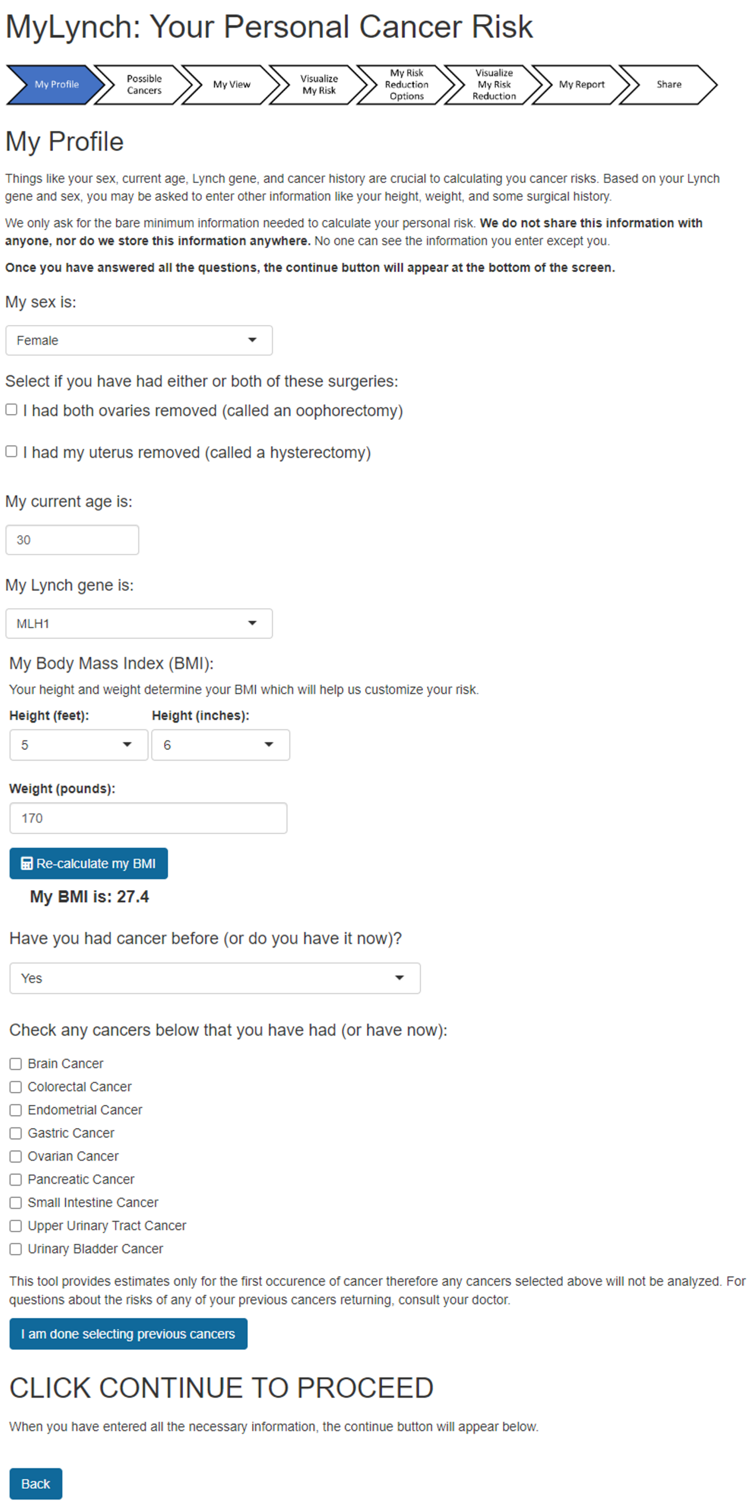

Figure S6: Screenshot of the page that collects the user's information with example values populated. The populated input values are for a hypothetical user. The information entered by the user is not stored anywhere and is not shared with anyone, other than whoever the user chooses to share the output report with. The information collected is the bare minimum required to personalize the user's cancer risks.

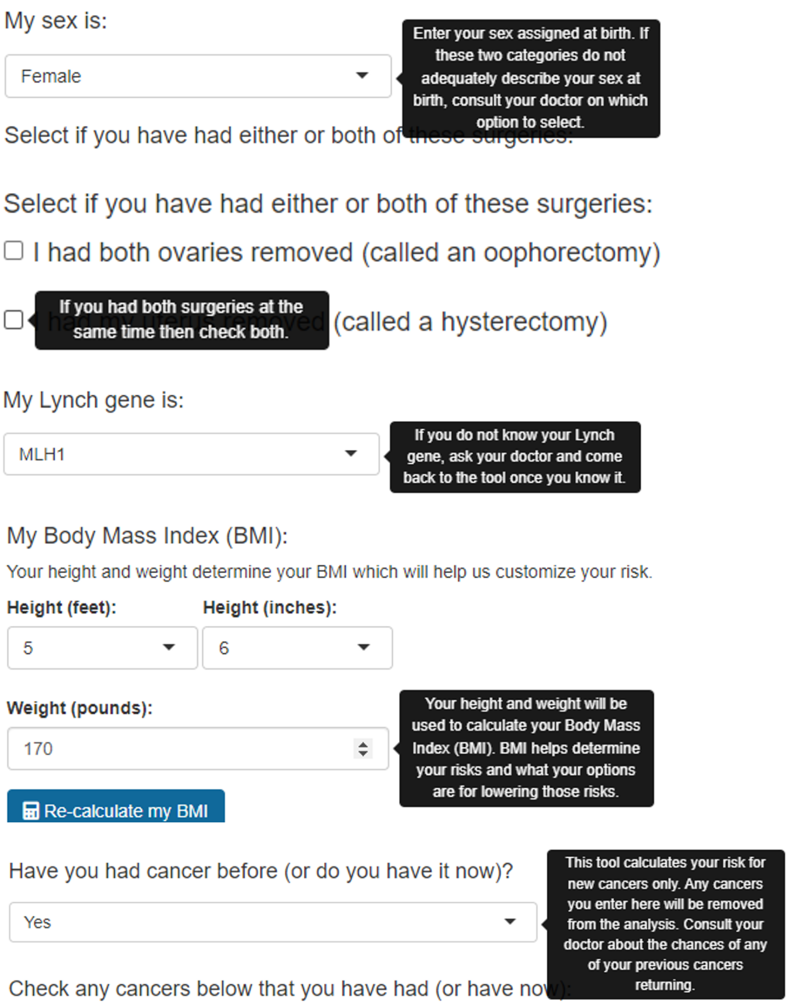

Figure S7: Screenshot of examples of some of the hover text on the screen which collects the user's information. The populated input values are for a hypothetical user. The text provides additional context related to the inputs. The information entered by the user is not stored anywhere and is not shared with anyone, other than whoever the user chooses to share the output report with.

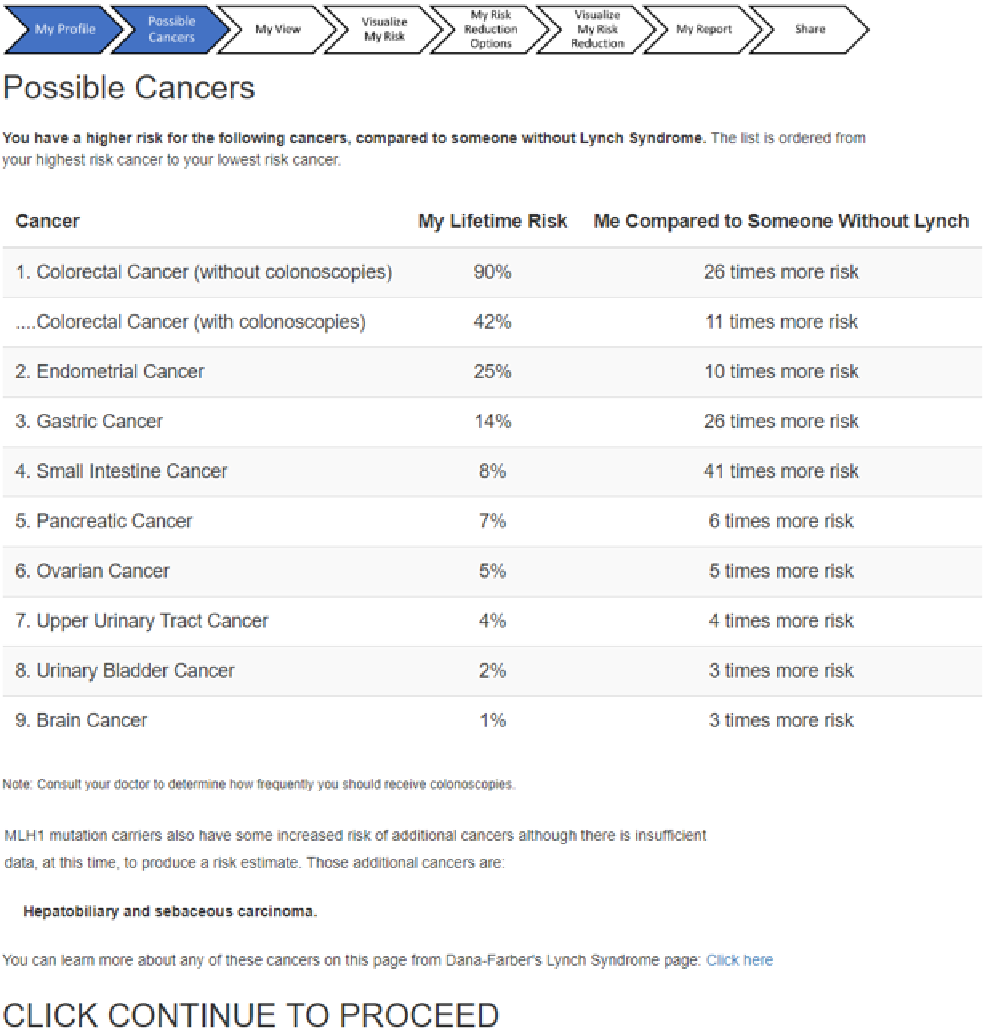

My Profile | Possible Cancers | My View | Visualize My Risk | My Risk Reduction Options | Visualize My Risk Reduction | My Report | Share

Possible Cancers

You have a higher risk for the following cancers, compared to someone without Lynch Syndrome. The list is ordered from your highest risk cancer to your lowest risk cancer.

Cancer	My Lifetime Risk	Me Compared to Someone Without Lynch
1. Colorectal Cancer (without colonoscopies)	90%	26 times more risk
....Colorectal Cancer (with colonoscopies)	42%	11 times more risk
2. Endometrial Cancer	25%	10 times more risk
3. Gastric Cancer	14%	26 times more risk
4. Small Intestine Cancer	8%	41 times more risk
5. Pancreatic Cancer	7%	6 times more risk
6. Ovarian Cancer	5%	5 times more risk
7. Upper Urinary Tract Cancer	4%	4 times more risk
8. Urinary Bladder Cancer	2%	3 times more risk
9. Brain Cancer	1%	3 times more risk

Note: Consult your doctor to determine how frequently you should receive colonoscopies.

MLH1 mutation carriers also have some increased risk of additional cancers although there is insufficient data, at this time, to produce a risk estimate. Those additional cancers are:

Hepatobiliary and sebaceous carcinoma.

You can learn more about any of these cancers on this page from Dana-Farber's Lynch Syndrome page: Click here

CLICK CONTINUE TO PROCEED

Figure S8: Screenshot of an example table of absolute and relative cancer risks presented to the user after they enter their information. Below the table, additional cancers are listed for which an association has been established in literature with a pathogenic mutation on the user's Lynch gene but, for which we lacked the data to establish baseline conditional penetrances. This example is customized to a hypothetical, obese, 30-year old female user with an unspecified race and ethnicity, a *MLH1* pathogenic mutation, no cancer history, and no history of relevant prophylactic surgeries.

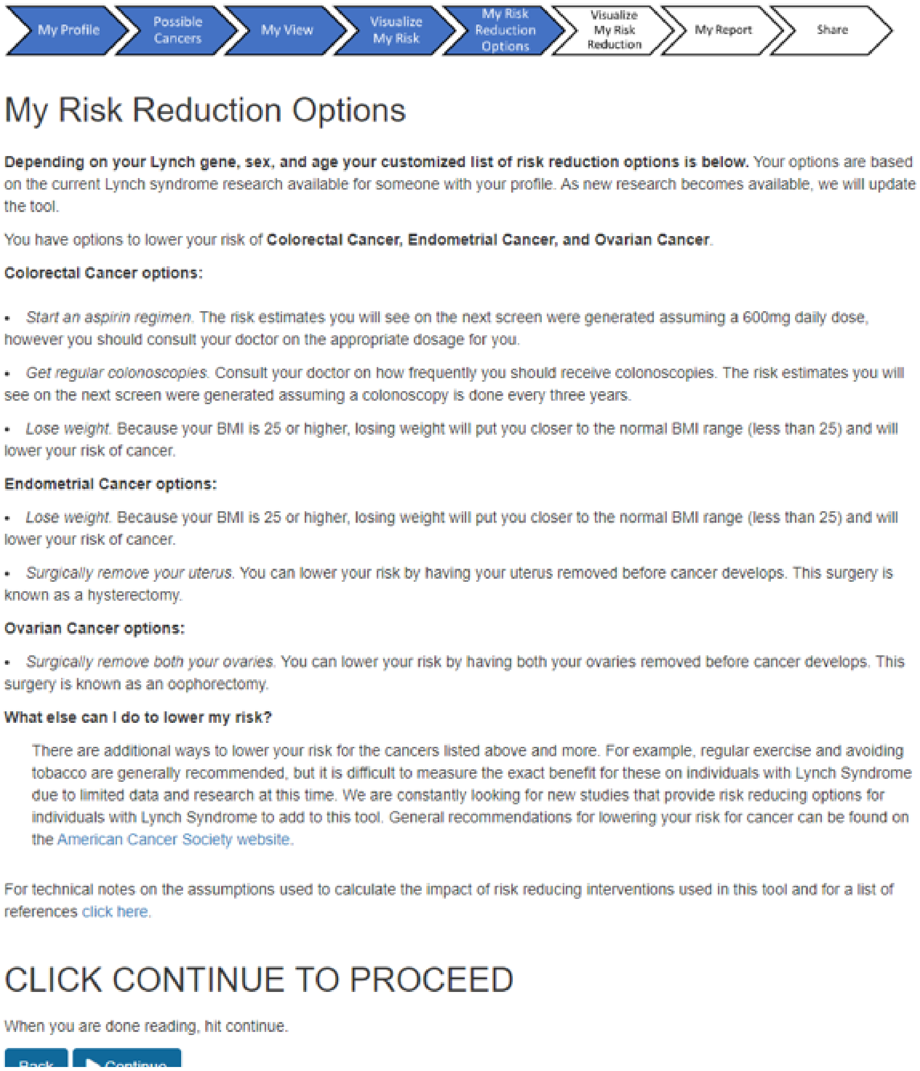

Figure S9: Screenshot of the page which explains the interventions available to the user, based on the information the user provided. This screen directly follows the screen which visualized the user's different cancer risks without the interactive intervention options. The interventions listed here are for a hypothetical, overweight or obese female user with no history of CRC, endometrial cancer, or ovarian cancer and who has undergone neither a hysterectomy nor oophorectomy.

Lynch Syndrome Cancer Risk Tool

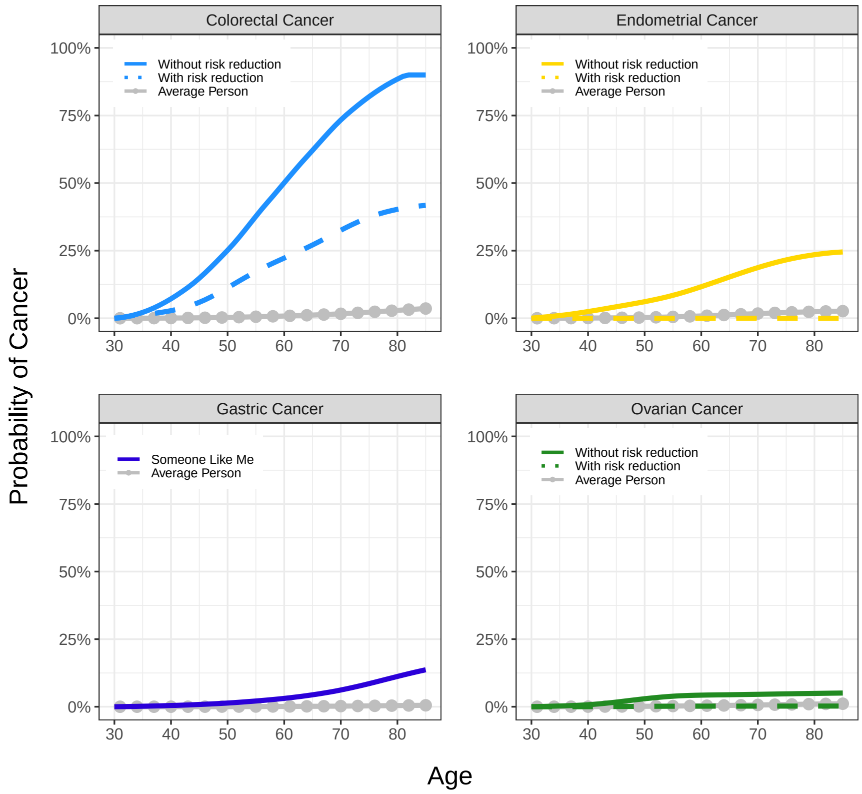

Dana-Farber Cancer Institute

What I Selected

Setting	Selected
Aspirin Regimen:	No
Colonoscopies:	Yes
Weight Loss:	Maintain current weight
New Oophorectomy:	Yes
New Hysterectomy:	Yes

Me

Setting	Selected
Sex:	Female
Age:	30
Lynch Gene:	MLH1
Height:	5ft 6in
Weight:	190lbs
Prior Oophorectomy:	No
Prior Hysterectomy:	No

Figure S10: Example of the personalized PDF report, customized to the user's selected cancers and interventions. In this example, the hypothetical user opted to include CRC, endometrial, gastric, and ovarian cancers on the report. The hypothetical user's information which the risk estimates were based on are shown in the table in the bottom right of the report and the interventions options the hypothetical user selected for the report are shown in the table in the middle right of the report. The report is sized for a 8.5x11 inch sheet of paper.

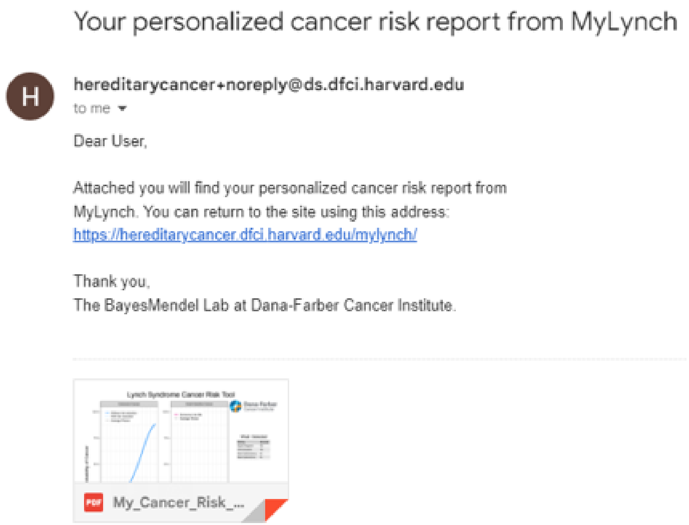

Figure S11: Screenshot of the email generated by MyLynch with a hypothetical user's personalized report as an attachment. The email is sent to the address provided by the hypothetical user. The email address and the PDF report are not stored or shared by MyLynch.

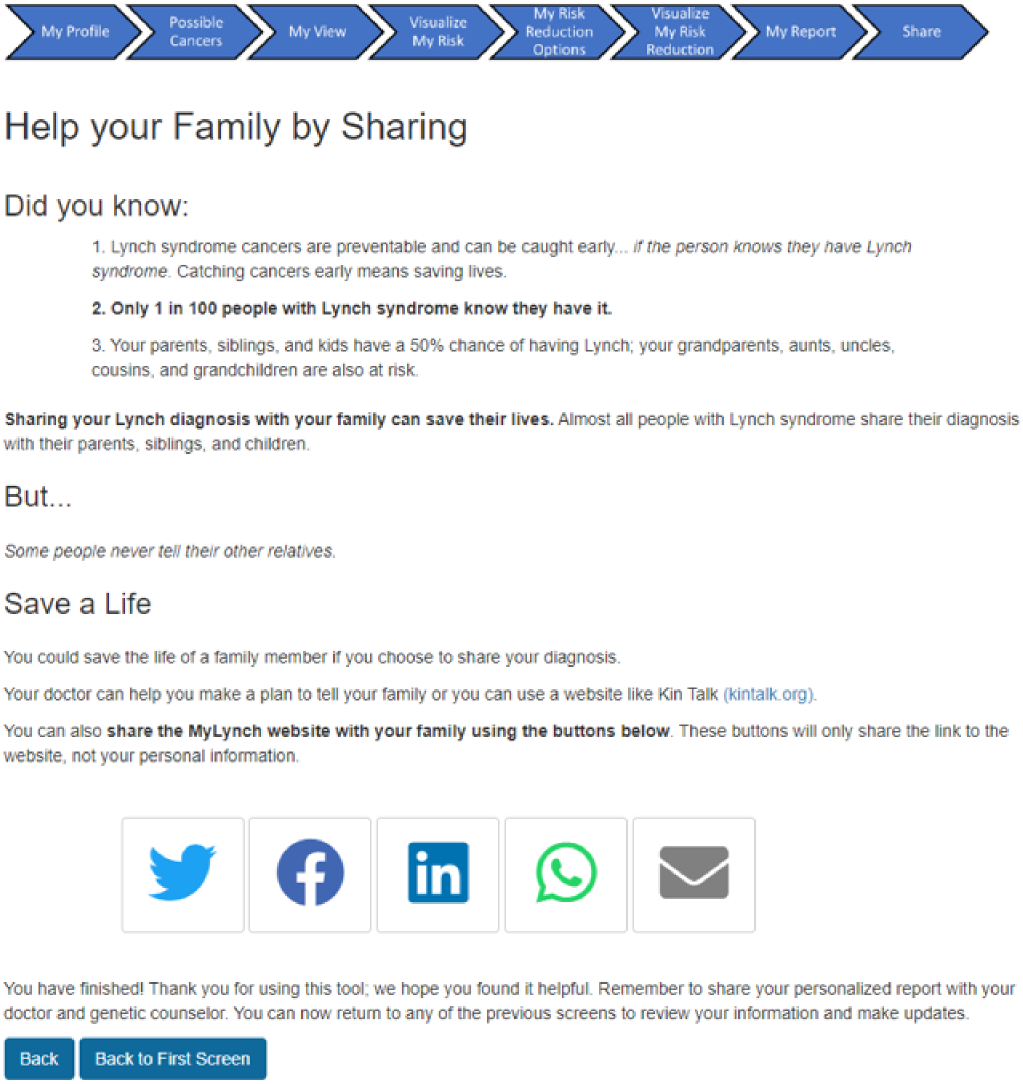

Figure S12: Screenshot of the last page of MyLynch which encourages users to share their LS diagnosis and the MyLynch web app with their family members.
